# Characterizing Daily-Life Social Interactions in Adolescents and Young Adults with Neurodevelopmental Disorders: A Comparison Between Individuals with Autism Spectrum Disorders and 22q11.2 Deletion Syndrome

**DOI:** 10.1007/s10803-021-05423-9

**Published:** 2022-01-11

**Authors:** Clémence Feller, Laura Ilen, Stephan Eliez, Maude Schneider

**Affiliations:** 1 Clinical Psychology Unit for Intellectual and Developmental Disabilities, Faculty of Psychology and Educational Sciences, University of Geneva, 40, Boulevard du Pont-d’Arve, 1205 Geneva, Switzerland; 2 Department of Neurosciences, Center for Contextual Psychiatry, KU Leuven, Leuven, Belgium; 3 Developmental Imaging and Psychopathology Lab Research Unit, Faculty of Medicine, University of Geneva, Geneva, Switzerland; 4 Department of Genetic Medicine and Development, Faculty of Medicine, University of Geneva, Geneva, Switzerland

**Keywords:** Ecological momentary assessment, Social interactions, Social functioning, Social phenotypes, Autism spectrum disorders, 22q11.2 deletion syndrome

## Abstract

Social impairments are common features of 22q11.2 deletion syndrome (22q11DS) and autism spectrum disorders (ASD). The Ecological Momentary Assessment (EMA) allowed access to daily-life information in order to explore the phenomenology of social interactions. 32 individuals with 22q11DS, 26 individuals with ASD and 44 typically developing peers (TD) aged 12–30 were assessed during 6 days 8 times a day using a mobile app. Participants with 22q11DS and ASD did not spend more time alone but showed distinct implication in the social sphere than TD. Distinct profiles emerged between the two conditions regarding the subjective experience of aloneness and the subjective experience of social interactions. This study highlights distinct social functioning profiles in daily-life in 22q11DS and ASD that points towards different therapeutic targets.

**Supplementary Information:**

The online version contains supplementary material available at 10.1007/s10803-021-05423-9.

## Introduction

Social interactions are at the very heart of our daily lives. Indeed, the desire to understand not only what social interactions entail but also what the individuals who participate in them think about these experiences is part of our social life. Adolescence, being a transition phase between childhood and adulthood is a highly relevant developmental period to investigate social interactions. In particular, it appears that socialization during adolescence, notably the growing emphasis on interactions with peers and the mounting complexity of social relationship (e.g. Zarrett & Eccles, 2006) as well as the emancipation from the family circle (Erikson et al., 1972), is crucial for the outcome of teenagers. Indeed, several studies have shown that social support during adolescence is important in mediating stress (e.g. MacKin et al., 2017), as well as for mental health outcomes and quality of life (e.g. Alsubaie et al., 2019; Hill et al., 2010; Thoits, 2007). Finally, the period of adolescence is also characterized by more conflictual social interactions (e.g. Brett, 1995). In order to fully understand social interactions during this period, it is important to focus not only on objective but also on subjective aspects. In this regard, recent studies (e.g. Achterhof et al., 2020) highlighted the importance of differentiating between objective and subjective aspects of interactions, namely “social behaviors”, that are quantifiable but that take away individual perspective (e.g. percentage of time spent alone), versus “social experiences” that are more experiential (e.g. how individuals subjectively experience their interactions with others). This distinction is relevant to consider since it has been found that subjective aspects of social interactions do not necessarily relate to objective aspects (Priebe & Fakhoury, 2008) and that subjective aspects might be more strongly related with outcome in adolescents (Achterhof et al., 2020).

If adolescence is a challenging time for social interactions in the general populations, they can be even more difficult for people with disabilities, and notably for individuals with neurodevelopmental conditions. The goal of the present study is to compare the social profile of individuals with 22q11.2 deletion syndrome (22q11DS), a neurogenetic condition affecting 1:2000–4000 live births that is associated with a broad phenotype of clinical and behavioral characteristics (McDonald-McGinn et al., 2017; Olsen et al., 2018), and Autism Spectrum Disorders (ASD). Individuals with 22q11DS present social functioning impairments in terms of adaptive behavior, social inhibition and isolation from peers (Schneider et al., 2014; Schonherz et al., 2014; Swillen, et al., 1997a, 1997b) that tend to become more pronounced during adolescence (Kates et al., 2015). In ASD, altered social interactions appear very early on and remain relatively stable or improve with age (e.g. Seltzer et al., 2004; Wallace et al., 2017), inducing difficulties in social reciprocity as well as in initiating and maintaining social interactions and relationships (Fakhoury, 2015; Jokiranta-Olkoniemi et al., 2016; Pugliese et al., 2015; Yang et al., 2016). These deficits in social functioning and social skills can lead to lower social participation and higher isolation that are associated with broad negative consequences and poor long-term outcomes (Lasgaard et al., 2010; Orsmond et al., 2013; Schneider et al., 2014; Seltzer et al., 2004; Wallace et al., 2017). ASD has been described as a frequent comorbidity of 22q11DS (e.g. Vorstman et al., 2006), although some studies also highlighted differences between the two conditions in the phenomenology of social impairments (Angkustsiri et al., 2014; Kates et al., 2007; McCabe et al., 2013). Therefore, these two conditions are particularly interesting to study together, with one of the goals of the present study being to disentangle what is common and what is different between the two conditions in terms of their social profiles.

Even if social impairments are widely acknowledged in these two groups, the majority of studies have used relatively general measures of social functioning, with more focus on “objective” aspects. Indeed, some studies point towards differences in *social behaviors* in individuals with ASD and in 22q11DS (e.g. Jawaid et al., 2012). For instance children with ASD spend half as much time with their peers compared to typically developing peers (TD) (Bauminger et al., 2003) and very few adults with ASD have had a long-term relationship (Hofvander et al., 2009). Individuals with 22q11DS have frequently been described as shy and more withdrawn from their peers than TD (e.g. Shprintzen, 2000; Swillen et al., 1997a, 1997b), and a study found that only few adults with 22q11DS were married, although half of these married couple had kids (Mosheva et al., 2018). However, less emphasis was placed on *social experiences*. Qualitative studies are an important source of information in this regard and highlight, for example, that individuals with ASD reported mixed experiences of being with friends, and many reported having experienced bullying (e.g. DePape & Lindsay, 2016). Despite these valuable insights, it is still unclear if individuals with ASD and 22q11DS like being alone or feel lonely (e.g. Deckers et al., 2017), and whether they spend more time alone because they lack interest to be with others or because they experience aversive feelings during interactions (e.g. Chevallier et al., 2012). It therefore appears of crucial importance to get a better understanding of the subjective experiences of social interactions in these populations.

As *social experiences* are ephemeral and context dependent (e.g. one may not experience a social interaction in the same way depending on whether it takes place with a familiar person or a stranger), it seems particularly necessary to find a way to observe how interactions are lived by people in real-life in the most naturalistic way. Henceforth, the Ecological Momentary Assessment (EMA), a structured diary technique that collects real-life measures in the everyday-life context (Myin-Germeys et al., 2009), appears to be a suitable technique to investigate social interactions in daily-life with high ecological validity. In practice, participants are notified on a given number of occasions during the day, at semi-random times, and are then asked to answer to a series of questions on smartphones or booklets. Moreover, EMA has several advantages, including multiple and in-the-moment assessments per day, which overcomes the problem of retrospective recall bias and the low correspondence that can be observed with paper–pencil questionnaires and interviews (Leendertse et al., 2018; Myin-Germeys et al., 2018; Schneider et al., 2017). It also captures fluctuation of the measured constructs such as emotional variability, and collects information in a natural setting that reflects real-life. Moreover, EMA gives access to the impact of context on affective states, which deepens the link between environmental context and its influence on internal states (Myin-Germeys et al., 2009, 2018). Of note, this method was found reliable to assess vulnerable populations, such as individuals with intellectual disability (Wilson et al., 2020) or schizophrenia (Hermans et al., 2020; Kwapil et al., 2020; Peters et al., 2012). To the best of our knowledge, this technique has only been used twice in adults with 22q11DS to characterize affective and psychotic reactivity to daily-life stress (Schneider et al., 2020; van Duin et al., 2019). In individuals with ASD, there is a paucity of studies (Chen et al., 2014, 2015, 2016, 2017; Chen, Bundy, et al., 2015; Chen, Cordier, et al., 2015; Chen, Cordier, et al., 2015; Cordier et al., 2014; Hintzen et al., 2010; Kovac et al., 2016; van der Linden et al., 2020), most of them focusing on the feasibility of the EMA technique. However, Hintzen and al. (2010) found that adults with ASD did not spend more time alone and did not report an increased preference for being alone compared to a control group, pointing toward a preserved desire to be in interaction with others. However, they expressed more negative affects when in the company of less familiar people compared to when they were alone, suggesting that social interactions outside of the direct family context can trigger negative affective states (Hintzen et al., 2010). In addition, Chen et al. (2015a), (2015b) highlighted that adolescents and adults were motivated to engage in social interactions in contexts where they felt competent and did not experience difficulties. Altogether, these results suggest a relatively preserved desire to interact with other people in individuals with ASD as well as a significant influence of the social context on affective states and motivation. However, these studies involved small samples (between 6 and 8 participants), highlighting the need to further investigate social experiences in daily life.

In the present study, EMA was used to characterize social functioning in the daily-life of adolescents and young adults with 22q11DS and ASD. Being alone or in the company of others as well as the subjective experience of these two contexts was investigated. In terms of *social behaviors*, we expected individuals with ASD and 22q11DS to spend more time alone than TD, more time with the persons they are living with, and less time with familiar persons not living with them as well as unfamiliar persons. In terms of *social experiences*, exploratory hypotheses were made because of the paucity of studies available in the literature. Therefore, we expected individuals with ASD and 22q11DS to report a different experience of aloneness (ExpA) and social interactions (ExpSI) compared to TD. Finally, we also expected that the social context (alone vs. with other people) would have a different influence on positive (PA) and negative affect (NA) in ASD and 22q11DS compared to TD.

## Method

### Sample

One hundred and seven participants (46% female) aged between 12 and 30 were included in the study (mean age = 18.54, SD = 4.28). Twenty-eight (46% female) individuals with ASD (mean age = 18.20, SD = 4.98) were recruited in clinical centers in Geneva and France, through a network of medical professionals and through announcements to family associations in Switzerland and France. Thirty-three (42% female) 22q11DS carriers (mean age = 19.19, SD = 4.67) were recruited through the 22q11DS Swiss longitudinal cohort which includes both Swiss and French individuals. Participants from the longitudinal cohort meeting inclusion criteria were asked to participate to an add-on study focusing on social difficulties, which included the EMA protocol described below as well as additional measures not described here. Forty-six (48% female) individuals were part of the TD group (mean age = 18.27, SD = 3.51) and were recruited through announcements at the University of Geneva and through the siblings of the 22q11DS individuals. Written consent was asked from caregivers for all participants with ASD and 22q11DS, as well as for TD under 18 years. This study was approved by the Swiss Ethics Committee on research involving humans (Commission Cantonale d’Ethique de la Recherche sur l’Etre Humain—CCER) in Geneva (CH).

Inclusion criteria for all participants were (1) age between 12 and 30 years and (2) sufficient command of the French language (fluid verbal communication) and sufficient reading abilities. All participants from the ASD group had a confirmed clinical diagnosis of ASD. They were assessed both with the Autism Diagnostic Observation Schedule, second version (ADOS-2;(Lord et al., 2012)), and their caregivers completed the Autism Diagnostic Interview-Revised (ADI-R;(Rutter, Le Couteur, et al., 2003)) or the Social Communication Questionnaire (SCQ;(Rutter, et al. 2003a, 2003b)). All participants from the 22q11DS group had a confirmed genetic diagnosis of microdeletion 22q11.2. They were screened with the Social Communication Questionnaire (SCQ; (Rutter et al. 2003a, 2003b)) with a mean score of 7.08. Six participants with 22q11DS had a score above the clinical cutoff (15). Participants with 22q11DS and ASD were assessed with through a semi-structured clinical interview to establish the presence of potential comorbid psychiatric disorders. Note that all participants were assessed with children or adults Wechsler intelligence scales (WISC-V;(Wechsler, 2014) or WAIS-IV;(Wechsler, 2011)) but intellectual deficiency was not an exclusion criterion since EMA can be used in populations presenting cognitive impairments (Wilson et al., 2020). For TD, exclusion criteria were (1) being born preterm, (2) having a first degree relative with any developmental disorder (siblings of participants with 22q11DS were included if the 22q11.2 deletion was confirmed to be de novo), (3) having a lifetime history of psychiatric (including neurodevelopmental disorders such as ASD), neurologic or learning impairments. Of note, TD were screened using the SCQ, with a mean score of 2.57 and none of the participants scoring above the clinical cutoff. The descriptive characteristics of the three groups are displayed in Table 1.

**Table 1:** Participant characteristics, psychiatric diagnosis and medication

		Diagnostic group				
		TD		22q11DS	ASD	
*N*		44		32	26	
Gender (female (%))		22 (50%)		13 (41%)	11 (42%)	
Age (mean (SD))		18.22 (3.41)		19.19 (4.75)	18.43 (5.06)	
Full Scale IQ (mean (SD))		111.3 (12.402)		71.35 (14.12)	108.35 (17.15)	
Current situation (%)						
School		97%		66%	82%	
Work		0%		28%	3%	
Unemployed		3%		6%	16%	
Psychiatric diagnosis (*N*(%))						
Simple phobia				5 (16%)	4 (15%)	
Agoraphobia				0	2 (8%)	
Social phobia				2 (6%)	4 (15%)	
Generalized anxiety				8 (25%)	3 (12%)	
Attention deficit disorder				6 (19%)	6 (23%)	
Oppositional defiant disorder				0	1 (4%)	
Mood disorders				4 (12%)	12 (46%)	
Psychosis				2 (6%)	0	
Obsessive–compulsive disorder				2 (6%)	1 (4%)	
ADOS-2 module 3 (*N* = 7) (mean)						
ADOS-2 total score					11.28	
ADOS-2 SA score					7.86	
ADOS-2 RRB score					3.43	
ADOS-2 module 4 (*N* = 19) (mean)						
ADOS-2 total score					12.47	
ADOS-2 SA score					9.42	
ADOS-2 RRB score					3.05	
ADI-R (*N* = 13) (mean)						
ADI-R domain A					17.15	
ADI-R domain B					12.69	
ADI-R domain C					5.62	
ADI-R domain D					2	
SCQ (*N* = 103) (mean)						
SCQ total score		2.57		7.08	17.12	
Medication						
Total (*N*(%))				24 (75%)	7 (27%)	
Categories						
Psychostimulant				9	1	
Antidepressants				14	3	
Neuroleptics				11	3	
Antiepilecptics				1	0	
Anxiolytics				4	2	

Comparison					
TD-22q11DS		TD-ASD		22q11DS-ASD	
Statistical test	*p*-value	Statistical test	*p*-value	Statistical test	*p*-value
*χ2* = .655	.418	*χ2* = .388	.533	*χ2* = 0.017	0.897
*F* = 1.051	.297	*F* = .044	.834	*F* = .355	.554
*F* = 158.532	**.000**	*F* = .753	.422	*F* = 79.817	**.000**

Significant p-values at the 0.05 level are displayed in bold

Typically developping peers (TD) had been screened for psychiatric diagnostics conforming to our exclusion criterias

Mood disorders include depressive disorder, dysthymia, bipolar disorder and severe mood dysregulation

Medication: total = number of participants under medication (some participants take more than one medication)

### Procedure and Assessment

The current research was carried out as part of a larger study also involving other tasks that are not described here. Participants received a voucher of 100 swiss francs (FNAC) or of 90 euros (amazon) for their participation to the study, regardless of their compliance to the EMA protocol. Participants, as well as their parents for ASD and 22q11DS participants and TD under 18 years old, were briefed about the EMA procedure. This EMA procedure was carried out through a mobile application (RealLife Exp) and its associated platform (lifedatacorp), that run on several operating systems. They are designed for the development of EMA studies so that the questionnaires (i.e. items, e.g. type of questions, and parameters, e.g. number of days) can be customized according to the study needs. The RealLife Exp app was then installed the participants’ smartphone and a trial questionnaire was completed with a member of the research team to obtain clarification if necessary. For younger participants and participants with cognitive difficulties, a trial questionnaire was also shown to the parents so that they could provide help if necessary (e.g. reminder of the meaning of a word). The EMA protocol was then carried out by the participants, with messages from the researchers every two days to verify study compliance and encourage participants. A descriptive report was sent to ASD and 22q11DS participants to give them feedback and suggest psychoeducational arrangements depending on their answers.

In the present study, the EMA protocol lasted for 6 days, with semi-random signal-contingent notifications 8 times per day between 7.30 AM and 10 PM. A minimum time window of 30 min was scheduled between two consecutive beeps. Participants had a maximum of 15 min to start completing the questionnaire, and an unlimited amount of time to fill out the questions. However, and this was not specified in the co-registration (osf.io/g4hv6), only the beeps that corresponds to a session length below 15 min were kept to ensure that participants’ answers correspond to the moment they were assessed (i.e. *in-the-moment* assessments). At each notification, the same momentary EMA questionnaire was delivered. It consisted of a minimum of thirty-three items and a maximum of thirty-eight items, depending on the answers to conditionally branched questions. There were no open-ended questions. PA (happiness, self-confidence, excitement, relaxation) and NA (sadness, anxiety, loneliness, anger) were assessed with a series of items, answered by a Likert scale ranging from 1 (not at all) to 7 (extremely). Participants then had to report whether they were alone (aloneness context) or in company of other persons (social context). They could report to be with up to three different types of company. Context was divided in four categories: (1) alone; (2) people they are living with (including pets); (3) familiar persons they don’t live with (family members; boyfriend/girlfriend; friends; colleagues/classmates); (4) unfamiliar persons (health professionals, acquaintances, strangers). When participants reported to be with several categories of people, the more familiar person was chosen (for instance if a participant reported to be with someone he was living with and a friend, we recoded it as “people they live with”: people they live with > familiar > unfamiliar). Of note, when individuals were living with their boyfriend or girlfriend, the category “someone I live with” was chosen. If participants reported to be alone, they were asked about their ExpA (aloneness appreciation, isolation and rejection feelings, desire to be with other people). On the contrary, they had to report their ExpSI (company appreciation, judgement and nervousness feelings, desire to be alone) when they were in company of other people. Of note, principal components analyses were performed to ensure that the items composing the different variables (i.e., PA, NA, ExpA and ExpSI) loaded on a single component (values above > 0.30). See Online Appendix 1 for EMA items and details about how they were aggregated into variables. In line with previous studies and general recommendations (Myin-Germeys et al., 2009; Palmier-Claus et al., 2011), only participants who answered to at least one-third of the beeps were kept in the analyses. A total of 5 participants were excluded from the analyses for this reason (n = 2 individuals with ASD, n = 1 individuals with 22q11DS, n = 2 TD). The final sample used for the analyses is therefore composed of 102 individuals (n = 26 ASD individuals with 880 valid notifications, n = 32 22q11DS individuals with 983 valid notifications, n = 44 TD with 1413 valid notifications).

### Statistical Analysis

Statistical analyses were conducted in STATA version 16.0. For all analyses, the level of statistical significance was set to p < 0.05. Analyses of variance (ANOVA) and chi-squared tests were used to investigated group differences in age, gender and IQ.

The data have a two-level structure: repeated measurements (level 1), nested within individuals (level 2). Multiple linear regression models were performed for group comparisons for time-invariant variables (*i.e.* one observation per participant, such as the percentage of time spent alone), using the REGRESS command. We controlled for age, gender and period of answers (*i.e.* holidays vs school/work). Note that we chose not to use IQ as a covariate since lower IQ is part of the phenotype of many neurodevelopmental disorders. Therefore, covarying for IQ would remove some of the variance inherent in the diagnosis (Dennis et al., 2009). Multilevel regression analyses were performed to compute group differences in time-varying variables (*i.e.* one observation per beep for participant, such as happiness). More specifically, mixed effects models with random intercepts were performed for group comparisons with the time-invariant categorical variable “group” used as a predictor and the time-varying continuous variables ExpA and ExpSI used as outcomes at the momentary level, using the XTSET/XTREG command. Mixed models with the time-invariant categorical variables “group”, “company” and “group*company” used as predictors and the time-varying continuous variables PA, NA and ExpSI used as outcomes at the momentary level were also performed to assess the impact of the different type of company, using the XTMIXED command. The B’s represent the fixed regression coefficients of the predictors in the multilevel model.

This study was co-registered on the OSF platform (osf.io/g4hv6) and the data are available open access on the Yareta preservation system. Two deviations from the original statistical analysis plan have to be noted. First, we decided to exclude some hypotheses that were initially pre-registered to increase the consistency of the study. Secondly, an additional analysis was included in this paper to examine the modulation of affect (PA and NA) by the context (alone vs. with others) and the group (22q11, ASD, and TD) as well as the context*group interaction. This was done using mixed models with the time-invariant categorical “group”, “context” and “group*context” used as predictors and the time-varying continuous affect variables PA and NA used as outcomes at the momentary level using the XTMIXED command. Finally, additional analyses were conducted to examine the effect of age on the appreciation of the context. Mixed models with the time-invariant categorical “group”, “age” and “group*age” used as predictors and the time-varying continuous variables experience of aloneness and experience of social interactions used as outcome at the momentary level were conducted using the XTMIXED command.

## Results

### Sample (EMA) Characteristics

The three groups were not statistically different in terms of age and gender but both participants with ASD (*F* (1, 55) = 79.817, *p* < 0.001) and TD (*F* (1, 68) = 158.532, *p* < 0.001) differed from 22q11DS on full-scale IQ scores. This was expected, given that impaired cognitive functioning is a core characteristic of individuals with 22q11DS. The average IQ level in the 22q11DS group was 71, which corresponds to what is typically reported in this population (e.g. Vorstman et al., 2015). The ASD group is mostly composed of individuals with average intellectual functioning (mean IQ = 108), with only 2 (8%) participants having an IQ in the intellectual disability range (IQ < 70). As mentioned in the methods section, 6 participants with 22q11DS scored above the clinical cutoff on the SCQ. To investigate the impact of participants with an elevated SCQ score on the obtained results, all the analyses were conducted while excluding these 6 participants and the results remained unchanged. The results reported below therefore include these 6 participants. Values are displayed in Table 1.

### Group Differences on Social Behaviors

There was no difference across the three groups regarding the percentage of time spent alone (22q11DS vs TD: (*b* = 0.634 (95% CI − 10.017 to 11.286), *p* = 0.906); ASD vs TD: (*b* = 5.715 (95% CI − 5.545 to 16.976), *p* = 0.316); 22q11DS vs ASD: (*b* = 5.372 (95% CI − 5.942 to 16.687), *p* = 0.345)).

Compared to the control group, participants with 22q11DS (*b* = 15.775 (95% CI 4.786–26.764), *p* = 0.005) and ASD (*b* = 23.483 (95% CI 4.866–24.101), *p* = 0.035) spent more time with people they live with compared to TD. There was no significant difference between the two clinical groups (*b* = − 3.057 (95% CI − 15.301 to 9.187), *p* = 0.619). Conversely, participants with 22q11DS (*b* = − 16.637 (95% CI − 26.072 to − 7.201), *p* = 0.001) and ASD (*b* = − 19.281 (95% CI − 29.356 to − 9.306), *p* = 0.000) both spent less time than TD with familiar individuals. There was no significant difference between the two clinical groups (*b* = − 312 (95% CI − 11.508 to 5.252), *p* = 0.457). To examine the different types of company within the category of familiar individuals, *post-hoc* analyses were conducted and revealed that 22q11DS and ASD individuals both spent less time with friends (22q11DS: (*b* = − 8.790 (95% CI − 15.529 to − 2.052), *p* = 0.011) ASD: (*b* = − 9.396 (95% CI − 16.520 to − 2.273), *p* = 0.010)) and with boyfriends/girlfriends (22q11DS: (*b* = − 9.477 (95% CI − 14.131 to − 4.824), *p* = 0.000) ASD: (*b* = − 7.101 (95% CI − 12.020 to − 2.181), *p* = 0.005)) than TD. However, there was no difference regarding the percentage of time spent in the company of relatives they don’t live with, nor in the time spent with classmates/colleagues (all p > 0.5). Finally, no statistical differences appeared between the three groups regarding the percentage of time spent with unfamiliar individuals (22q11DS vs. TD (*b* = 0.231 (95% CI − 1.910 to 2.372), *p* = 0.831); ASD vs. TD (*b* = 0.966 (95% CI − 1.297 to 3.229), *p* = 0.399); 22q11DS vs ASD: (*b* = 0.683 (95% CI − 1.932 to 3.319), *p* = 0.598)). The variables of interest’s mean values are displayed in Table 2.

**Table 2:** EMA items (mean, SD))

Variables		TD	22q11DS	ASD	Results
Percentage of time spent alone (mean (SD))		38.017 (23.159)	41.234 (25.125)	44.738 (24.036)	ASD = 22q11DS = TD
Percentage of time spent with people they are living with (mean (SD))		27.471 (27.991)	39.974 (26.350)	39.152 (26.415)	ASD = 22q11DS, 22q11DS & ASD > TD
Percentage of time spent with familiar people (mean (SD))		32.094 (25.756)	16.135 (19.374	12.663 (12.032)	ASD = 22q11DS, 22q11DS & ASD < TD
	Friend	11.608 (13.448)	1.861 (3.778)	4.463 (8.686)	ASD = 22q11DS, 22q11DS & ASD < TD
	Classmate/colleage	4.737 (8.736)	7.297 (15.567)	4.341 (6.696)	ASD = 22q11DS = TD
	Boyfriend/girlfriend	9.281 (21.961)	1.110 (3.133)	0 (0)	ASD = 22q11DS, 22q11DS & ASD < TD
	Family	6.465 (12.373)	5.866 (12.088)	3.858 (8.281)	ASD = 22q11DS = TD
Percentage of time spent with unfamiliar people (mean (SD))		2.335 (4.190)	2.577 (4.201)	3.253 (5.549)	ASD = 22q11DS = TD
Positive affects (PA) (mean (SD))	Overall	4.038 (.680)	4.034 (1.024)	3.468 (.837)	ASD < TD, ASD = 22q11DS, 22q11DS = TD
	Alone	3.955 (.690)	4.349 (2.638)	3.224 (.671)	ASD < 22q11DS, ASD < TD, 22q11DS = TD
	In company	4.081 (.742)	3.799 (1.202)	3.880 (2.517)	ASD < TD, ASD = 22q11DS, 22q11DS = TD
Negative affects (NA) (mean (SD))	Overall	1.509 (.461)	1.795 (1.108)	2.153 (1.009)	ASD < TD, ASD = 22q11DS, 22q11DS = TD
	Alone	1.631 (.544)	2.105 (1.199)	2.244 (1.144)	ASD > 22q11DS, ASD = TD, 22q11DS = TD
	In company	1.492 (1.019)	1.649 (1.046)	2.147 (.833)	ASD > TD, ASD = 22q11DS, 22q11DS = TD
Experience of aloneness (ExpA)		2.472 (.878)	2.646 (.785)	2.540 (1.060)	ASD = 22q11DS = TD
	Preference to be with others	2.478 (1.436)	2.700 (1.330)	2.203 (1.239)	ASD = 22q11DS = TD
	Like to be alone_reverse	3.841 (1.513)	3.763 (1.464)	3.519 (1.679)	ASD = 22q11DS = TD
	Feeling excluded	1.097 (.239)	1.476 (.924)	1.897 (1.207)	ASD > TD, ASD = 22q11DS, 22q11DS = TD
Experience of social interactions (ExpSI)		1.647 (.365)	1.913 (.629)	2.561 (.755)	ASD < 22q11DS, ASD < TD, 22q11DS = TD
	Preference to be alone	1.527 (.516)	2.209 (1.295)	2.655 (1.100)	ASD = 22q11DS, ASD < TD, 22q11DS < TD
	Like company_reverse	2.756 (.737)	2,773 (.951)	3.756 (1.135)	ASD < 22q11DS, ASD < TD, 22q11DS = TD
	Feeling nervous	1.154 (.309)	1.369 (.769)	2.028 (.898)	ASD < 22q11DS, ASD < TD, 22q11DS = TD
	Feeling judged	1.153 (.256)	1.301 (.514)	1.805 (.713)	ASD < 22q11DS, ASD < TD, 22q11DS = TD

### Group Differences on Social Experiences

#### Experience of Aloneness (ExpA)

Regarding ExpA, no significant difference was found between the three groups (TD vs ASD (b = 0.052 (95% CI − 0.390 to 0.495), p = 0.818); TD vs 22q11DS (b = 0.142 (95% CI − 0.284 to − 0.390), p = 0.512); ASD vs. 22q11DS (b = − 0.092 (95% CI − 0.587 to 0.403), p = 0.715). However, when comparing the groups on the individual items composing ExpA, we observed that individuals with ASD felt more excluded than TD (b = 0.755 (95% CI 0.357 to 1.152), p = 0.000), but not than 22q11DS (b = 0.495 (95% CI − 0.064 to 1.054), p = 0.083). There was no difference between TD and 22q11DS (b = 0.285 (95% CI − 0.098 to 0.669), p = 0.145). No difference appeared in the preference to be with others between the groups (TD vs 22q11DS (b = 0.203 (95% CI − 0.440 to 0.846), p = 0.536), TD vs 22q11DS (b = − 0.275 (95% CI − 0.941 to 0.390), p = 0.536), 22q11DS vs ASD (b = − 0.493 (95% CI − 1.177 to 0.190), p = 0.157)). Finally, there was no difference in the appreciation of aloneness between the groups (TD vs 22q11DS (b = − 0.050 (95% CI − 0.781 to 0.679), p = 0.892), TD vs 22q11DS (b = − 0.322 (95% CI − 1.080 to 0.434), p = 0.404), 22q11DS vs ASD (b = − 0.286 (95% CI − 1.127 to 0.555), p = 0.505)). The variables of interest’s mean values are displayed in Table 2. Note that adding IQ as a covariate did not change the results (data not shown). Additional analyses showed that age had no effect on the experience of aloneness overall, and group * age interactions were also not significant (all p > 0.05).

#### Experience of Social Interactions (ExpSI)

Participants with ASD reported a significantly worse subjective appreciation of social interactions (ExpSI) than both TD (*b* = 0.863 (95% CI 0.596 to 1.130), *p* = 0.000) and participants with 22q11DS (*b* = 0.660 (95% CI 0.321 to 0.999), *p* = 0.000). TD did not differ from 22q11DS (*b* = 0.208 (95% CI − 0.045 to 0.461), *p* = 0.107). Looking at each item composing ExpSI individually, participants with ASD reported feeling significantly more judged than both TD (*b* = 0.605 (95% CI 0.378 to 0.832), *p* = 0.000) and participants with 22q11DS (*b* = 0.503 (95% CI 0.212 to 0.794), *p* = 0.001). TD did not differ from 22q11DS (*b* = 0.106 (95% CI − 0.109 to 0.322), *p* = 0.333). They also reported to feel more nervous in the company of other people compared to both TD (*b* = 0.831 (95% CI 0.531 to 1.130), *p* = 0.000) and participants with 22q11DS (*b* = 0.737 (95% CI 0.333 to 1.141), *p* = 0.000). TD did not differ from 22q11DS (*b* = 0.101 (95% CI − 0.182 to 0.386), *p* = 0.482). Participants with ASD also rated the company of people they were with to be less pleasant than both TD (*b* = 0.923 (95% CI 0.489 to 1.358), *p* = 0.000) and participants with 22q11DS (*b* = 0.940 (95% CI 0.424 to 1.456), *p* = 0.000). TD did not differ from 22q11DS (*b* = -0.011 (95% CI − 0.423 to 0.401), *p* = 0.957). Finally, individuals with ASD (*b* = 1.086 (95% CI 0.617 to 1.554), *p* = 0.000) and 22q11DS (*b* = 0.637 (95% CI 0.193 to 1.081), *p* = 0.005) both reported that they would prefer to be alone while with others to a greater extent than TD. The two clinical groups did not differ from each other regarding this specific item (*b* = 0.453 (95% CI − 0.178 to 1.085), *p* = 0.160). Overall, participants did not report a change in ExpSI depending on the company type (*b* = − 0.172 (95% CI − 0.364 to 0.021), *p* = 0.081). Moreover, the ExpSI*company type interaction was not significant, indicating that the impact of the company type on ExpSI was similar between the groups (all p > 0.5). The variables of interest’s mean values are displayed in Table 2. Note that adding IQ as a covariate did not change the results (data not shown). Additional analyses showed that age had no effect on the experience of social interactions overall (p > 0.05) but the group*age interaction was significant, indicating that the association between age and experience of social interactions differ across groups (22q11DS vs. TD (*b* = − 0.208 (95% CI − 0.040 to -0.001), *p* = 0.041), 22q11DS vs TSA (*b* = 0.174 (95% CI 0.000 to 0.349), *p* = 0.050) TSA vs TD (*b* = 0.032 (95% CI 0.010 to 0.054), *p* = 0.004). Indeed, in TD group (*b* = − 0.012 (95% CI − 0.024 to 0.001), *p* = 0.056) there was a significant negative association between age and ExpSI that was stronger than in the 22q11DS group (*b* = − 0.011 (95% CI − 0.025 to − 0.004), *p* = 0.174). In the ASD group, the association between age and ExpSI was positive (*b* = 0.035 (95% CI 0.011 to 0.058), *p* = 0.004).

### Influence of Context on Affects

On average, participants with ASD reported more NA overall than TD (*b* = 0.578 (95% CI − 0.205 to 0.952), *p* = 0.002), regardless of the context. Participants with 22q11DS did not differ from ASD (*b* = 0.358 (95% CI − 0.142 to 0.861 0.952), *p* = 0.161) nor from TD (*b* = 0.231 (95% CI − 0.122 to 0.584), *p* = 0.199) on NA level. All participants reported more NA when alone compared to when in company of other people (*b* = 0.160 (95% CI 0.956 to 0.263), *p* = 0.002). However, the group*context interaction was not significant (all p > 0.05), indicating that the association between NA and the social context was similar in the three groups. Overall, participants did not report a change in NA depending on the company type (*b* = − 0.034 (95% CI − 0.171 to 0.103), *p* = 0.628). Moreover, the group*company type interaction was also not significantly associated with NA, indicating that the type of company did not influence NA differently in the three groups (all p > 0.05). Mean levels of NA are displayed in Table 2.

On average, participants with ASD reported less PA overall than TD (*b* = − 0.611 (95% CI − 1.034 to − 0.188), *p* = 0.005), regardless of the context. Participants with 22q11DS did not differ from ASD (*b* = − 0.491 (95% CI − 0.994 to 0.012), *p* = 0.056) nor from TD (*b* = − 0.114 (95% CI − 0.514 to 0.285), *p* = 0.574) on PA level. Participants did not report a change in PA when alone compared to when in company of other people (*b* = − 0.099 (95% CI − 0.211 to 0.021), *p* = 0.081). However, the group*context interaction approached significance between TD and 22q11DS (*b* = 0.174 (95% CI 0.000 to 0.349), *p* = 0.050), indicating that individuals with 22q11DS reported significantly higher PA when alone and lower PA when in company of others, the opposite pattern being observed in TD. ASD did not differ from TD, nor from 22q11DS (p > 0.05). Overall, participants did not report a change in PA depending on the company type (*b* = 0.146 (95% CI − 0.054 to 0.347), *p* = 0.154). The group*company type interaction was not significantly associated with PA, indicating that the type of company did not influence PA differently in the three groups (all p > 0.05). Mean levels of PA are displayed in Table 2.

## Discussion

This is the first study to characterize social functioning in daily-life in a relatively large sample of adolescents and young adults with ASD and 22q11DS using EMA. Our main findings indicate that both participants with ASD and 22q11DS show similar *social behaviors.* In particular, they spent a comparable amount of time alone, more time with the people they live with but less time with familiar individuals (*e.g. *friends) than TD. Overall, participants with ASD and 22q11DS also reported a similar experience of aloneness (*ExpA*), with the exception that individuals with ASD reported feeling more excluded than both participants with 22q11DS and TD. By contrast, they reported markedly different social experiences (*ExpSI*), with individuals with ASD reporting worse ExpSI than both participants with 22q11DS and TD. The only similarity between the two clinical groups in terms of ExpSI was a higher desire to be alone when in company of other people compared to TD. Regarding *the influence of context on affect*, individuals with ASD reported less PA and more NA than the other two groups, regardless of the context. Finally, being in the company of other people had a beneficial impact on affect in TD, whereas this benefit was less clear in 22q11DS.

### Social Behaviors

The present study challenges the commonly accepted idea that social withdrawal is a characteristic of neurodevelopmental disorders (Bauminger et al., 2003; Jawaid et al., 2012; Schneider et al., 2012; Schonherz et al., 2014; Seltzer et al., 2004; Wallace et al., 2017), as the three groups reported spending a similar amount of time alone (*i.e.* physical absence of other people or *aloneness* (Lay et al., 2019)). This is however in line with the findings of Hintzen and al. (2010), who also used EMA, whereas the remaining studies used more classical approaches to measure social withdrawal, such as questionnaires and interviews. Moreover, the majority of studies used information reported by caregivers and not by the participants themselves. That being said, even if the present study highlights a comparable amount of time spent alone between the three groups, it also suggests that individuals with 22q11DS and ASD have a different involvement in the social world compared to TD. Indeed, they both reported spending more time with the people they live with and less time in the company of familiar persons outside of the direct family circle, friends in particular. However, a similar amount of time spent with classmates and colleagues was reported between the groups. This is in line with previous studies reporting smaller social networks in individuals with ASD (Howlin et al., 2004; Kasari et al., 2011; Locke et al., 2010; Orsmond et al., 2004, 2013) and the fact that individuals with 22q11DS have been described to be more isolated from peers (Schonherz et al., 2014). These results also highlight the central role of the family environment in the lives of adolescents and young adults with neurodevelopmental disorders (Gulec-Aslan et al., 2013; e.g. Seltzer et al., 2000) and suggest that emancipating from the family circle appears to be a challenge for youth with 22q11DS and ASD.

In line with previous reports (e.g. Hauck et al., 1995), these results could suggest that individuals with 22q11DS and ASD have fewer opportunities of interactions, especially in less structured environments, which may prevent them from broadening their social network. Indeed, participants with 22q11DS and ASD reported spending a comparable amount of time with classmates or colleagues than their peers, which is probably explained by the fact that these interactions mostly take place in relatively structured environment (*e.g. *school or work). These interactions might therefore be more predictable and more accessible than that one can have with friends. Of note, the majority of our sample was composed of participants who were still attending school, which ensures at least a minimal number of social encounters through these structured contexts. This lack of opportunity to interact with peers, especially in less structured settings, may be related to the fact that social initiatives are more difficult for youth with ASD and 22q11DS. Incidentally, adolescents with ASD were found to rely more on parents to facilitate social relationships (Bauminger et al., 2003; Howard et al., 2006; Lasgaard et al., 2010), which would mean that they spend time with family members in order to access time with friends. In the present study, the different types of company were computed to be mutually exclusive. For this reason, we could not identify if participants reported to be in the company of both a family member and a friend, which could support the idea of family as a way of accessing friends. Moreover, cognitive level was found to account for spontaneous initiations of interaction with peers (Bauminger et al., 2003). Particularly in the group of participants with 22q11DS, whose average intellectual functioning level is in the borderline range, this could contribute to explain the differences in the time spent with friends. Indeed, interactions with friends mostly take place in less structured contexts and therefore rely more on the ability to actively initiate an interaction and less on the ability to follow well defined “social scenarios”. It should be acknowledged that the observed group differences in terms of *social behaviors* could also result from a different labeling of their social environment. It is possible that when TD refer to people as “friends”, individuals with ASD and 22q11DS refer to them as “classmates/colleagues”. Indeed, this distinction requires a deep and accurate understanding of the different types of relationships as well as a certain introspection to fully gasp the distinction between the different kind of relationships (Bauminger & Kasari, 2000). Distinguishing between friends and acquaintances was also found to be difficult for many individuals with ASD in a qualitative study (Carrington et al., 2003). This is in line with the precited hypothesis of a misunderstanding of the different degree of friendship and the derived terms (*e.g. *friend or classmate).

In summary, the present study shows that individuals with 22q11DS and ASD are not characterized by social withdrawal as such but that social interactions take place much more within the restricted family circle and in relatively structured environments. Since most of our participants were still attending school—which provides opportunities for structured social interactions—the results of the present study suggest that the transition after school and towards independent living should also be anticipated from a social perspective in order to avoid a decrease in the number of social contacts. A longitudinal follow-up of such a cohort would provide a unique opportunity to investigate how social behaviors evolve during this transition period from adolescence to adulthood.

### Social Experiences

#### Experience of Aloneness (ExpA)

Contrary to our expectations, the three groups reported a similar subjective experience of aloneness. This is a major finding considering that social disinterest is typically considered to be a feature of both ASD and 22q11DS (e.g. Jawaid et al., 2012; Morel et al., 2018). Taken together, the results of this study suggest that individuals with 22q11DS and ASD are not characterized by social withdrawal from an objective point of view (*i.e.* social behavior) but also do not report social disinterest from a subjective point view (*i.e.* social experiences). Moreover, it is particularly interesting to note that the three groups did not differ on the item “I’d rather be with other people”, pointing toward a preserved motivation for interpersonal interactions, as pointed out by previous studies (Bauminger et al., 2003; Deckers et al., 2014; Maddox & White, 2015). It should be noted that the findings discussed in the context of the present study are based on group comparisons that may mask substantial interindividual variability. Future studies should aim to parse this heterogeneity in order to identify relevant subgroups of individuals characterized by distinct social profiles. Such an approach has recently been employed by Uljarevic et al. (2020) who used a social functioning questionnaire to cluster individuals with ASD based on their social phenotype. However, given the limitations of classical measures of social functioning to assess social experiences in an ecologically valid way (Schneider et al., 2017), future studies may use EMA to identify more relevant subgroups.

Despite an overall similar subjective experience of aloneness among the three groups, individuals with ASD reported higher levels of exclusion and isolation feelings than both participants with 22q11DS and TD when looking at individual items composing ExpA one by one, which points towards increased loneliness (*i.e*. a negative emotional experience (Lay et al., 2019)) in this population. These findings are in line with previous studies that found higher levels of loneliness in younger (Bauminger et al., 2003; Bauminger & Kasari, 2000; Lasgaard et al., 2010; e.g. Locke et al., 2010; White & Roberson-Nay, 2009) and older adolescents with ASD (e.g. Deckers et al., 2017; Mazurek, 2014; Sundberg, 2018), though none of them used EMA to measure this construct. As suggested by Maddox and al. (2015), the subjective feeling of social isolation experienced by individuals with ASD could be explained by a lack of knowledge about how to form relationships. Besides, as adolescence is a transition phase during which individuals experience new relationships and the expectations towards these relationships evolve (Qualter et al., 2013), loneliness can appear when there is a gap between the expectations and reality (Heinrich & Gullone, 2006; Lay et al., 2019). Interestingly, one of the few studies that directly compared individuals with ASD and 22q11DS found higher levels of empathy, sense of humor and other complex social skills in 22q11DS than in idiopathic ASD (Angkustsiri et al., 2014), elements that could possibly play a role in preventing them to feel lonely. Indeed, if individuals with ASD have little access to these complex social skills, it could lead to a worse comprehension of social interactions, and therefore to feelings of rejection and a greater experience of loneliness. Of note, social anxiety was also found to be related to greater loneliness (Danneel et al., 2018; Eres et al., 2020; Lim et al., 2016; Maes et al., 2019; Reed et al., 2016; White & Roberson-Nay, 2009), and this comorbidity was reported twice as much in our sample of individuals with ASD compared to the 22q11DS group, playing a plausible role in explaining the distinct experience of aloneness between the two conditions. Moreover, social anxiety was described by Hintzen et al. (2010) as the discrepancy between the fear of rejection and the desire for social interaction, a pattern that matches what is reported by individuals with ASD in the present study. Future studies should aim to better investigate loneliness in individuals with ASD and with 22q11DS by taking in account the potential impact of social anxiety.

### Experience of Social Interactions (ExpSI)

Contrary to the experience of aloneness, participants with ASD reported a markedly different—and more negative—experience of social interactions than both individuals with 22q11DS and TD, providing information about *how* social interactions are experienced in daily-life and not only about the quantity of social interactions. This finding is not consistent with the previous report of Hintzen and al. (2010), who observed that individuals with ASD mostly enjoyed the company of other people. This could be explained by the fact that our sample, being younger than the one of Hintzen and al. (mean age = 28.3), is characterized by less mature emotion regulation strategies. Indeed, the latter are known to improve with age (Zimmermann & Iwanski, 2014), and in younger sample like ours, could contribute to the rather negative experience of social interactions. Additionally, emotion regulation difficulties have been shown to be inherent to ASD and contribute to the socioemotional and behavioral problems they experience (e.g. Mazefsky et al., 2013). For instance, experiences of bullying and peer victimization were reported to be particularly frequent among individuals with ASD during adolescence (e.g. Sterzing et al., 2012). Moreover, additional analyses revealed a positive relationship between age and ExpSI, with younger participants with ASD reporting poorer experiences of social interactions than older participants, which is consistent with the above-mentioned hypotheses.

Of particular interest, the present study highlights that the subjective experience of social interactions is markedly different between individuals with ASD and those with 22q11DS, with subjective reports of ExpSI in the 22q11DS group being similar to those of TD. Previous findings suggests that individuals with both 22q11DS and ASD are characterized by social anhedonia (Milic et al., 2021; Novacek et al., 2016; Tan et al., 2020), described as a diminished social interest and a lack of pleasure from social contact leading to withdrawal (Brown et al., 2007). However, the present study offers new insights regarding the subjective experience of social interactions that challenge this assumption. As already stated above, these discrepancies might arise from the fact that previous studies used parent-reported information collected in a laboratory setting, whereas the present study uses self-reported information collected in the daily-life of individuals. In our sample of participants with ASD, the profile of answers was more characteristic of social anxiety than of a diminished social interest. Indeed, they reported a negative experience of social interactions and a lower enjoyment of the company of others compared to both participants with 22q11DS and TD, but did not spend more time alone. The reports of participants with 22q11DS during social interactions were also not indicative of a diminished social interest, as they experienced social interactions rather positively and rated the pleasantness of their social company similarly to TD. When alone, they also reported wanting to be with other people to the same extant than TD, which is also suggestive of a preserved motivation to interact with others. Of note, ExpSI was not influenced by the type of company in any of the three groups, suggesting that the profile described above reflects how participants experienced their social interactions *in general*. However, it should be noted that the small number of occurrences during which participants were in company of unfamiliar people prevented us from examining ExpSI in this social context specifically. In line with previous studies (e.g. Hintzen et al., 2010), it is likely that they would have resulted in a more negative ExpSI.

Interestingly, both participants with ASD and 22q11DS reported a greater preference for being alone when they were in the company of other people, regardless of the type of company. Incidentally, this was the only significant difference between individuals with 22q11DS and TD, who otherwise reported a similar positive appreciation of social interactions. This could possibly be explained by the cost of interacting: for individuals with ASD or 22q11DS, social interactions might require greater efforts than for TD, hence the higher desire to be alone, although social interactions were not as unpleasant for 22q11DS as they were for ASD. In line with this interpretation, being in the company of other people had a beneficial impact on affective states in the TD group. Indeed, higher levels of NA and lower levels of PA were observed when alone, the oppositive pattern being found when in the company of others. This is in line with a study using EMA that found more happiness and interest, as well as less sadness, pain and tiredness when individuals were engaged in social interactions as opposed to when they were not (e.g. Bernstein et al., 2018). In individuals with 22q11DS, this benefit was less clear since the social context had a similar impact on NA than in TD—highlighting the positive impact of being with others on NA—but an opposite impact on PA. Higher PA when alone could be explained by the fact that, spending a lot of time home, participants with 22q11DS didn’t necessarily always choose to be interacting with the people they live with, which could explain why they report greater PA when alone and a higher wish to be alone when in company. Moreover, interactions probably required greater effort for participants with 22q11DS, which could contribute to making them feel more relaxed and joyful when alone.

Altogether, the results regarding social behaviors and the subjective experience of social interactions suggest that individuals with 22q11DS and ASD could benefit from different therapeutic intervention targeting social impairments, which is an interesting avenue for future investigations.

### Strengths, Limitations and Future Directions

This is the first study comparing the social phenotype in daily-life of two neurodevelopmental conditions—ASD and 22q11DS. By using EMA, contextual information is taken into account and offers more granulated information that consider daily variations, therefore ensuring that participants’ answers reflect their actual environment accurately. Moreover, it shows feasibility of this method in neurodevelopmental disorders, reinforcing EMA literature that is still scare in this domain. Furthermore, the present study contributes to better distinguish the social profile of these two conditions often considered to be partially overlapping.

However, results of the present study should be considered in light of several methodological limitations. First, EMA relies on participants subjective self-report. Although one member of the research team went over EMA items with all the participants, interpretation may still differ from one individual to another. Moreover, as the 22q11DS group had a significantly lower IQ than both ASD and TD, the level of comprehension could have been different between the groups. Of note, Wilson and al. (2020) validated EMA feasibility in a population with moderate intellectual disability but this technique had rarely been used in 22q11DS population (Schneider et al., 2020; van Duin et al., 2019). This is why we took time to go through the protocol with each participant and to carefully read and explain each item, as well as closely monitoring them during the full EMA period. Being available for questions and technical issues experimented by participants also had an impact on study compliance, since only 5 participants were excluded from the original sample because of an insufficient number of answered beeps.

Secondly, heterogeneity within the 22q11DS and ASD groups should be considered. Indeed, various comorbidities and medications were present in both clinical groups, possibly having an impact on the results. However, comorbidities are more the rule than the exception in neurodevelopmental disorders (e.g. De Smedt et al., 2007; Thapar et al., 2017). Given the variety of comorbidities reported, it wasn’t possible to subdivide our groups accordingly but future studies should aim to further investigate this important question. Six participants with 22q11DS scored above the clinical cutoff on the SCQ, suggesting concerns for a potential diagnosis of ASD (the presence of an ASD diagnosis was not formally examined in this group). To examine the influence of these participants on the obtained results, all the analyses were conducted while excluding these participants and the results remaining unchanged. This suggests that the results obtained in the 22q11DS group are not explained by the presence of comorbid autistic traits in a subgroup of participants. Another limitation of the current study is that the ASD group is mostly composed of individuals with average intellectual functioning, with only 8% of the ASD group presented a comorbid intellectual disability. For this reason, the obtained results cannot be extended to individuals with lower intellectual functioning. However, unlike previous EMA research in ASD (Chen et al., 2015, 2016; Chen, Bundy, et al., 2015; Chen, Cordier, et al., 2015; Chen, Cordier, et al., 2015; Cordier et al., 2014; van der Linden et al., 2020; Wilson et al., 2020), individuals with ASD regardless of the intellectual functioning level were recruited and not only individuals with average intellectual functioning. Third, the cultural impact was not investigated in this study. Future studies should investigate factors related to the cultural background of participants that may influence their evaluations of the social context. Finally, alexithymia wasn’t examined in the present study. Given the high prevalence of alexithymia among individuals with ASD (Milosavljevic et al., 2016), it would have been useful to get a deeper comprehension on how this could influence their answers.

## Conclusions

The present study showed comparable *social behaviors* in ASD and 22q11DS, with an increased percentage of time spent with people they live with and less time spent with familiar people than TD. However, the two clinical groups did not differ from TD on the amount of time spent alone, challenging the commonly accepted assumption that neurodevelopmental disorders are characterized by social withdrawal. Regarding *social experiences*, the results of the present study point towards distinctive social phenotypes in ASD and 22q11DS, with a more negative experience of social interactions and greater loneliness in individuals with ASD, and a more positive experience of social interactions among individuals with 22q11DS. Therefore, even if adolescents and young adults with ASD and 22q11DS individuals are similar from an objective perspective (*i.e.* social behaviors), they differ from each other on a subjective view (*i.e.* social experiences), which emphasizes the need to develop specific intervention targets in the two populations.

## Supplementary Information

Below is the link to the electronic supplementary material.

Supplementary file1 (docx 16 KB)

## Data Availability

The data set is publicly available through the YARETA data preservation system.

## Abbreviation

22q11DS: 22q11.2 deletion syndrome
ASD: autism spectrum disorders
HC: healthy controls
ExpA: experience of aloneness
ExpSI: experience of social interactions
EMA: Ecological Momentary Assessment
PA: positive affect
NA: negative affect
CCER: Commission Cantonale d’Ethique de la Recherche sur l’Etre Humain
ADI-R: Autism Diagnostic Interview-Revised
ADOS-2: Autism Diagnostic Observation Schedule, second version
SCQ: Social Communication Questionnaire
WAIS-IV: Wechsler Adult Intelligence Scale, fourth edition
WISC-V: Wechsler Intelligence Scale for Children, fifth edition
