# Supplementary material for: Characterizing Daily-Life Social Interactions in Adolescents and Young Adults with Neurodevelopmental Disorders: A Comparison Between Individuals with Autism Spectrum Disorders and 22q11.2 Deletion Syndrome
Source: J Autism Dev Disord. 2022 Jan 11;53(1):245–62. doi: 10.1007/s10803-021-05423-9 (PMC9889413; doi:10.1007/s10803-021-05423-9)
Supplement: Supplementary file 1 — Supplementary file1 (docx 16 KB) [file 10803_2021_5423_MOESM1_ESM.docx]

**Appendix 1.** EMA items used to compute variables

**1.** The variable positive affect (PA) was calculated on the average score of 4 items, all rated on a 7-point Likert scale ranging from 1 to 7. PCA was performed to ensure that the components load on the construct (>.30). Mean scores of the 4 items was then taken to compute the PA value, with high scores representing higher positive affect. The items are:

- "I feel happy, joyful"

- "I feel excited"

- "I feel relaxed"

- "I trust myself"

**2.** The variable negative affect (NA) was calculated on the average score of 4 items, all rated on a 7-point Likert scale ranging from 1 to 7. PCA was performed to ensure that the components load on the construct (>.30). Mean scores of the 4 items was then taken to compute the negative affects value, with high scores representing higher negative affect. The items are:

- "I feel sad"

- "I feel lonely"

- "I feel anxious, worried"

- "I feel irritated, angry"

**3.** The variable context (alone versus in company) was made from a branched question “Are you alone?” answered by “yes” or “no”.

**a.** Non-social context corresponds to the item "I am alone" answered 1.

**b.** Social context corresponds to the item "I am alone" answered 0. Multiple choices then include:

- "someone I live with"

- "a family member I don't live with"

- "my boyfriend/girlfriend"

- "a friend"

- "a classmate/colleague"

- "an acquaintance"

- "a pet"

- "a stranger"

Context was then subdived in four categories:

1) alone

2) people they are living with, composed of the answers "someone I live with" and "a pet"

3) familiar persons they don't live with, composed of the answers "family members I don't live with", "boyfriend/girlfriend", "friends", "colleagues/classmates"

4) unfamiliar persons, composed of the answers "health professional", "acquaintance", stranger"

**4.** The variable experience of aloneness (ExpA) was based on the subjective appreciation of the non-social context (i.e., only when participants reported to be alone). The mean of the following items was used:

- "I like being alone" (reversed score for analyses)

- "I feel isolated or excluded"

- "I'd rather be with other people".

These items were also rated on a 7-point Likert scale ranging from 1 to 7. PCA was performed to ensure that the components load on the construct (>.30). Mean scores of the 3 items were taken to compute the experience of aloneness variable, with higher scores representing worse experience of aloneness.

**5.** The variable experience of social interactions (ExpSI) was based on the subjective appreciation of the social context (i.e., only when participants reported to be in company of other people). The mean of the following items was used:

- "The company is pleasant" (reversed score for analyses)

- "I feel judged by this/these person/people"

- "I am nervous in this/these person/people company"

- "I'd rather be alone".

These items were also rated on a 7-point Likert scale ranging from 1 to 7. PCA was performed to ensure that the components load on the construct (>.30). Mean scores of the 4 items were taken to compute the experience of social interactions variable, higher scores representing worse experience of social interactions.
